# Unveiling DNA damage repair-based molecular subtypes, tumor microenvironment and pharmacogenomic landscape in gastric cancer

**DOI:** 10.3389/fgene.2023.1118889

**Published:** 2023-04-14

**Authors:** Weiqi Kong, Zhiqiang Wang, Bingyi Wang

**Affiliations:** Department of General Surgery, Tongren Hospital, Shanghai Jiao Tong University School of Medicine, Shanghai, China

**Keywords:** gastric cancer, DNA damage repair, clinical outcomes, tumor microenvironment, immunotherapy, pharmacogenomics

## Abstract

**Objective:** The current molecular classification system for gastric cancer covers genomic, molecular, and morphological characteristics. Non-etheless, classification of gastric cancer based upon DNA damage repair is still lacking. Here, we defined DNA damage repair-based subtypes across gastric cancer and identified clinicopathological, tumor microenvironment and pharmacogenomic features.

**Methods:** Unsupervised clustering analysis was executed in the TCGA-STAD cohort based upon the transcriptional expression profiling of DNA damage repair genes. LASSO computational approach was adopted for generating a DNA damage repair-relevant gene signature. The identified subtypes or signature were externally verified in the GSE84426 or GSE84433 cohort. The transcriptional levels of immunomodulators, abundance of immune cells and somatic mutations were measured, respectively. Immunotherapeutic response, and drug sensitivity were investigated. The DNA damage repair-relevant genes were further experimentally verified.

**Results:** Two DNA damage repair-based subtypes were identified, with the notable heterogeneity in prognostic stratification, tumor microenvironment and somatic mutations. The gene signature was generated for risk stratification and prognostic prediction, which was in relation to immunomodulators and immune cells. High-risk cases were more likely to respond to immunotherapy, with distinct pharmacogenomic landscapes between low- and high-risk groups. Higher levels of PAPPA2, MPO, MAGEA11, DEPP1, CPZ, and COLEC12 and lower level of CYTL1 were proven in gastric cancer cells *versus* controls. Silencing CYTL1 facilitated intracellular ROS accumulation and suppressed migration in gastric cancer cells.

**Conclusion:** Collectively, the DNA damage repair-based classification is a suitable complement to existing molecular classification system, and the quantitative gene signature provides a robust tool in selecting specific therapeutic options.

## Introduction

Gastric cancer remains a major contributor to global cancer incidence and mortality, which is responsible for over 700,000 deaths each year (Sung et al., 2021). More than 95% of gastric cancer cases are adenocarcinomas, usually classified according to anatomical location and histological type (Ajani et al., 2022). Among individual patients, gastric cancer usually exhibits extensive histological, transcriptomic and epigenomic variations, with varying clinical behaviors and therapeutic responses (Sheng et al., 2021). Incorporating the interpatient heterogeneity into clinical management and determining the molecular features that drive gastric cancer variations is a key strategy to improve patients’ survival (Wei et al., 2022). Despite advance in defining specific molecular subtyping of gastric cancer in TCGA and ACRG consortia, *etc.*, the actual improvement in prognosis based upon them is modest, with increasing understanding that gastric cancer also exhibits high interpatient heterogeneity (Cancer Genome Atlas Research Network, 2014; Cristescu et al., 2015). Therefore, further exploration of molecular heterogeneity features within gastric cancer patients is required for understanding the critical principles that govern clinical outcomes and management for gastric cancer (Qiu et al., 2020).

DNA damage can arise from endogenous or exogenous sources, while DNA repair is required to maintain genome integrity. In addition to direct repair, DNA damage response system comprises numerous pathways: base excision repair, mismatch repair, nucleotide excision repair, homologous recombination repair, non-homologous end joining, *etc.* (Jiang et al., 2021). Abnormal DNA damage repair exerts a key implication in tumor initiation and malignant development (Li et al., 2021). Moreover, tumors with impaired DNA repair machinery may display higher genomic instability, thus driving malignant development, and producing a more aggressive tumor phenotype (Liu et al., 2021). For example, PAICS results in gastric carcinogenesis and is involved in DNA damage response *via* interaction with histone deacetylase 1/2 (Huang et al., 2020). The cellular efficiency of DNA damage repair mechanisms also exerts an important role in therapeutic response of gastric cancer (Liu et al., 2021). For instance, Bcl-2-associated transcription factor 1 Ser290 phosphorylation modulates DNA damage response and radiotherapy resistance in gastric cancer (Liu et al., 2021). Targeting Chk2 improves gastric cancer chemotherapeutic effects through impairing DNA damage repair (Gutiérrez-González et al., 2013). Evidence also demonstrates the implication of DNA damage repair in immunotherapy, e.g., inducing a higher tumor mutation burden (TMB) that generates more neoantigens, thereby promoting immunological surveillance and tumor-infiltrating lymphocyte infiltrations (Rizvi et al., 2015). Given the importance of DNA damage repair, we proposed a novel DNA damage repair-based subtyping as a suitable complement to existing molecular classification system of gastric cancer, and a quantitative gene signature as a robust tool to aid in selecting appropriate therapeutic options, which might facilitate the development of precision medicine.

## Materials and methods

### Gastric cancer cohorts

RNA sequencing data, clinical information, and somatic mutation data of TCGA-STAD (*n* = 370) were acquired from the GDC (https://portal.gdc.cancer.gov/). Counts data were normalized to CPM values through edgeR package (Robinson et al., 2010). Microarray expression profiling and clinical information were gathered from the GSE84426 (*n* = 76) and GSE84433 (*n* = 357) (Yoon et al., 2020) on the Illumina platform, used for external verification.

### Collection of DNA damage repair genes

Totally, 449 DNA damage repair genes were gathered from the Molecular Signatures Database (http://www.broadinstitute.org/msigdb), which were listed in Supplementary Table S1 (Liberzon et al., 2015).

### Unsupervised clustering analysis

Unsupervised clustering approach based upon Euclidean and Ward’s linkage was adopted for determining molecular subtypes in accordance with the transcriptional levels of DNA damage repair genes. ConsensusClusterPlus package was implemented for identifying the optimal number of clusters according to consensus cumulative distribution function (CDF) across TCGA-STAD patients (Wilkerson and Hayes, 2010). Principal component analysis (PCA) was conducted for proving the distribution difference between subtypes. Kaplan–Meier (K-M) curves were plotted for comparing overall survival (OS) of distinct subtypes, followed by log-rank test. Then, conventional clinicopathological parameters were compared between subtypes. The subtyping classification was externally verified in the GSE84426 dataset.

### Gene set enrichment analysis (GSEA)

The up- or downregulated Gene Ontology (GO) or Kyoto Encyclopedia of Genes and Genomes (KEGG) terms in two groups were analyzed through adopting GSEA software (Subramanian et al., 2005). The reference gene sets were acquired from the GO (Ashburner et al., 2000) and KEGG (Kanehisa and Goto, 2000) databases.

### Somatic mutation analysis

Somatic mutation data from TCGA-STAD dataset were acquired utilizing TCGAbiolinks package (Colaprico et al., 2016), and mutation annotation format was analyzed and summarized with maftools package (Mayakonda et al., 2018). TMB was also computed, which was defined as the somatic mutation number per megabase of interrogated genomic sequence (Sha et al., 2020).

### Immune infiltration analysis

The fraction scores of 22 immune cell types were inferred utilizing CIBERSORT computational method (Newman et al., 2015).

### Differential expression analysis

Genes with differential expression were explored between the DNA damage repair-based subtypes utilizing edgeR package. The criteria were set as |fold change (FC)|≥2 and adjusted *p* < 0.05.

### Construction of a DNA damage repair-relevant gene signature

For revealing the prognostic implication of DNA damage repair, prognosis-related DNA damage repair-relevant genes with *p* < 0.01 were selected for least absolute shrinkage and selection operator (LASSO) analysis utilizing glmnet package (Engebretsen and Bohlin, 2019). Genes with non-zero coefficients were chosen with ten-fold cross-validation. TCGA-STAD samples were randomly classified as training and test datasets with a ratio of 1:1. Meanwhile, GSE84433 dataset was adopted as external verification. Thereafter, a DNA damage repair-relevant gene signature was generated, following the formula: $RiskScore=\underset{i}{\sum }Coefficient\;of\;gene\;\left(i\right)*expression\;of\;gene\;\left(i\right)$ . All cases were classified as low- and high-risk groups through the median RiskScore. K-M curves of OS were conducted, and 1-, 3-, and 5-year receiver operating characteristic (ROC) curves were plotted with timeROC package. Uni- and multivariate Cox regression approaches were utilized for examining whether the RiskScore acted as an independent prognostic parameter.

### Immunotherapy response prediction

Immunotherapy response was inferred through Tumor Immune Dysfunction and Exclusion (TIDE) algorithm based upon two major tumor immune escape mechanisms: triggering T cell dysfunction in tumor tissue with highly infiltrated cytotoxic T lymphocytes (CTLs) and preventing the infiltration of T cells into tumor tissue with lowly infiltrated CTLs (Jiang et al., 2018). High TIDE score indicates a greater possibility of anti-tumor immune evasion, thus exhibits a low immunotherapy response. Transcriptome data and survival information of patients who received immunotherapy were gathered from the GSE78220 (Hugo et al., 2016), GSE91061 (Riaz et al., 2017) and IMvigor210 (Necchi et al., 2017) cohorts.

### Drug sensitivity estimation

Expression matrix and drug processing information were obtained from the Cancer Genome Project 2014, and IC50 values of anti-cancer drugs were estimated with pRRophetic package (Geeleher et al., 2014).

### Cell culture

Human gastric mucosa epithelial cell line GES-1 and human gastric cancer cell lines HGC-27, MKN-28 and AGS were provided by Procell (China). All cells were cultivated in RPMI-1640 medium (Gibco, United States) plus 10% fetal bovine serum, 100 IU/mL penicillin and 100 mg/mL streptomycin at 37°C with 5% CO_2_.

### RT-qPCR

Total RNA was extracted utilizing Trizol (Solarbio, China), with 5 µg for cDNA synthesis. RT-qPCR was executed through SYBR Premix Ex Taq kit (Takara, China) together with Step-one real-time PCR system (ABI, United States). The mRNA level was standardized to Tubulin. The primers were synthesized by Sangon (China) (Table 1). Relative mRNA level was computed based upon 2^−ΔΔCT^ approach.

**TABLE 1:** The information of primers for RT-qPCR.

Gene name	Sequence (5′-3′)
PAPPA2	F: AGA​ATA​AGC​CTG​GCG​ATT​TTG​G
	R: GGC​CTT​AGG​TAG​TTC​CCA​GC
MPO	F: TGC​TGC​CCT​TTG​ACA​ACC​TG
	R: TGC​TCC​CGA​AGT​AAG​AGG​GT
MAGEA11	F: TCC​CAG​GAT​CTG​CCA​AGA​GTC
	R: CCC​CAC​AGC​ACT​TGT​TCT​C
DEPP1	F: TGC​CCA​CAA​TTC​GGG​AGA​C
	R: AGA​CCT​CAC​GTA​GTC​ATC​CAG
CPZ	F: CTG​CTG​GTC​ATC​GAG​TTC​TCC
	R: TGC​CAC​CTC​ATA​GCC​GTC​A
COLEC12	F: AAT​CCT​TCG​GTT​ACA​AGC​GGT
	R: ACT​GTG​ATT​GTT​AGC​AAG​GCA​C
CYTL1	F: ATG​TGT​GAG​ATA​CCT​GCC​CAG
	R: CAA​GGC​ATT​GCA​GTC​ATC​CAA
Tubulin	F: TGG​ACT​CTG​TTC​GCT​CAG​GT
	R: TGC​CTC​CTT​CCG​TAC​CAC​AT

Abbreviation: F, forward; R, reverse.

### Immunoblotting

RIPA lysis (Solarbio) was adopted for extracting total protein, and protein samples electrophoresed onto polyacrylamide gels were loaded onto PVDF membranes that were then blocked utilizing 5% BSA for 1 hour. The membranes were probed with primary antibody of PAPPA2 (PA5-21046; ThermoFisher, United States), MPO (1/1000; ab208670; Abcam, United States), MAGEA11 (1/500; ab96236), DEPP1 (1/1000; ab230977), CPZ (15944-1-AP; Proteintech, China), COLEC12 (1/1000; ab278081), CYTL1 (1/500; ab129767) or Tubulin (1/5000; ab7291) at 4°C in a shaker overnight. Incubation with HRP goat anti-rabbit IgG (1/10,000; ab288151) for 1 hour, followed by development. ImageJ software was adopted for grayscale analysis.

### Cell transfection

Small interfering RNAs (siRNAs) targeting CYTL1 (RiboBio) were transfected into cells based upon Lipofectamine RNAiMax (Life Technologies) in accordance with the manufacturer’s protocols.

### Detection of intracellular reactive oxygen species (ROS)

In accordance with the manufacturer’s protocols (RiboBio), intracellular ROS was measured utilizing ROS fluorescent probe (Dihydroethidium). Under a fluorescence microscope (Olympus, Japan), images were investigated and photographed.

### Wound healing assay

Cells were planted into 6-well plates. Thin scratches were created utilizing a sterile pipette tip. Under an inverted microscope (Olympus), images were photographed immediately (0 h) and marked the 6-well plate so that the same field could be located again. After incubation for 24 h, the culture medium was removed and the cells were washed for removing surrounding cellular debris.

### Statistical analysis

All statistical analyses were executed through R language (version 4.2.1). Two groups with normally distributed variables were compared with Student’s *t*-test, with Wilcoxon test for non-normally distributed variables. One-way ANOVA was conducted for comparing ≥3 groups. Pearson or Spearman correlation test was utilized for estimating the association between variables. *p* < 0.05 was regarded as statistical significance, as denoted: *p < 0.05; **p < 0.01; ***p < 0.001; ****p < 0.0001.

## Results

### Construction and verification of a novel DNA damage repair-based subtype classification of gastric cancer

**FIGURE 1:**
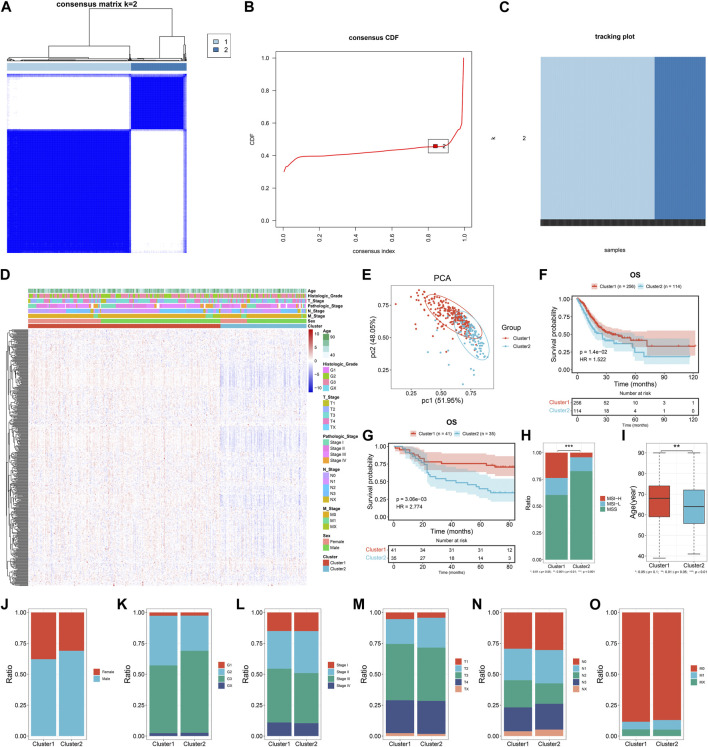
Construction and verification of a subtype classification of gastric cancer based on DNA damage repair genes. **(A–C)** Consensus matrix, CDF, and track plot across TCGA-STAD based upon the expression values of DNA damage repair genes. **(D)** Transcriptional levels of DNA damage repair genes in the two DNA damage repair-based subtypes. Colors from blue to red denote low to high expression levels. **(E)** PCA for proving the subtype classification according to the expression values of DNA damage repair genes. **(F,G)** K-M curves of OS between subtypes in the TCGA-STAD and GSE84426 cohorts. **(H)** Distribution of MSI-L, MSI-H and MSS subtypes across the two subtypes in the TCGA-STAD dataset. **(I–O)** Distribution of clinicopathological traits across the two subtypes in the TCGA-STAD dataset.

The current study gathered 449 DNA damage repair genes, and according to their expression values, TCGA-STAD samples were classified as DNA damage repair activating and inhibitory subtypes called cluster1 and cluster2 through adopting unsupervised clustering method (Figures 1A–C). In contrast to cluster2, most DNA damage repair genes displayed high expression in cluster1, demonstrating the DNA damage repair activating and inhibitory status of cluster1 and cluster2 (Figure 1D). PCA proved the prominent difference in two subtypes based upon the transcription levels of DNA damage repair genes (Figure 1E). In Figure 1F, we investigated the difference in OS outcomes between subtypes, with better OS for cluster1. This DNA damage repair-based subtyping was externally verified in the GSE84426 cohort. As expected, cluster1 exhibited the notable advantage in OS compared with cluster2 (Figure 1G). Afterwards, we assessed clinicopathological traits of two subtypes in the TCGA-STAD cohort. TCGA project has classified gastric cancer into four major subtypes. Among them, microsatellite instability (MSI) subtype often comprises genetic and/or epigenetic silence of mismatch repair genes. As shown in Figure 1H, cluster1 had the higher ratios of high MSI (MSI-H), and low MSI (MSI-L) as well as the lower ratios of microsatellite stable (MSS) in comparison to cluster2. Patients in cluster1 had older age in contrast to those in cluster2 (Figure 1I). In addition, it was found that higher ratios of male cases and more advanced histological grade were observed in cluster2 (Figures 1J, K). However, we did not observe the notable differences in pathological stage, and TNM stage between subtypes (Figures 1L–O).

### Signaling pathways underlying the DNA damage repair-based subtypes

**FIGURE 2:**
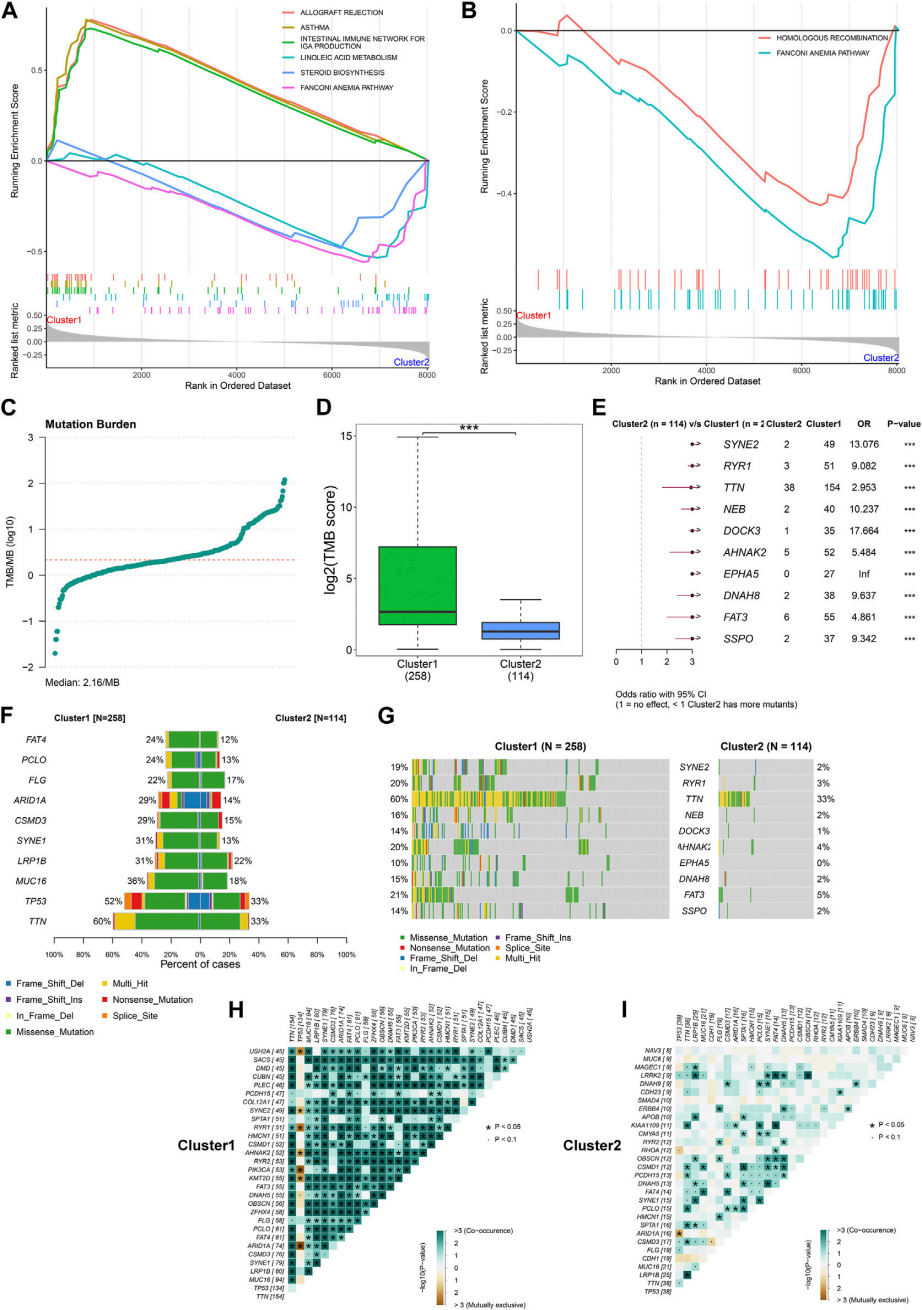
Differences in signaling pathways and somatic mutation between the two DNA damage repair-based subtypes across TCGA-STAD. **(A)** GSEA for the enrichment levels of signaling pathways in the two subtypes. For the enrichment score line chart, the horizontal axis denotes the sorted genes, and the vertical axis denotes the corresponding running enrichment score. The peak in the line graph is the enrichment score of the gene set, and the gene before the peak is the core gene under the gene set. In the middle of the panel, lines mark genes under that gene set. The lower part of the panel shows the distribution of rank values for all genes. **(B)** GSEA for the enrichment levels of DNA damage repair pathways in the two subtypes. **(C)** Distribution of TMB score across TCGA-STAD. **(D)** Comparison of TBM score between subtypes. **(E)** Odd ratio (OR) of mutated genes between subtypes. OR>1 denotes that cluster1 has more mutants than cluster2. **(F,G)** The frequency of the top ten mutated genes across the two subtypes. **(H,I)** Relationships between mutated genes in cluster1 and cluster2, respectively.

Next, mechanisms underlying the two DNA damage repair-based subtypes were analyzed through GSEA. Consequently, Cluster1 exhibited higher activity of immune-related pathways (allograft rejection, asthma, and intestinal immune network for IgA production), with higher activity of linoleic acid metabolism, steroid biosynthesis and Fanconi anemia pathway in Cluster2 (Figure 2A). Thereafter, relative enrichment levels of DNA damage repair pathways were compared between subtypes. Intriguingly, homologous recombination and Fanconi anemia pathway were mainly enriched in Cluster2 (Figure 2B).

### Somatic mutation difference between the two DNA damage repair-based subtypes

We gather somatic mutation data from the TCGA-STAD dataset, and computed TMB score. Figure 2C depicts the distribution of TMB across TCGA-STAD samples, with the median value of 2.16/MB. In contrast to cluster2, higher TMB score was observed in cluster1 (Figure 2D). SYNE2, RYR1, TTN, NEB, DOCK3, AHNAK2, EPHA5, DNAH8, FAT3, and SSPO occurred more frequent mutations in cluster1 (Figures 2E–G). In addition, we observed the co-occurrence of mutated genes in each subtype. Intriguingly, there was more extensive co-occurrence of mutation in cluster1 in contrast to cluster2 (Figures 2H, I).

### Tumor microenvironment heterogeneity across the two DNA damage repair-based subtypes

**FIGURE 3:**
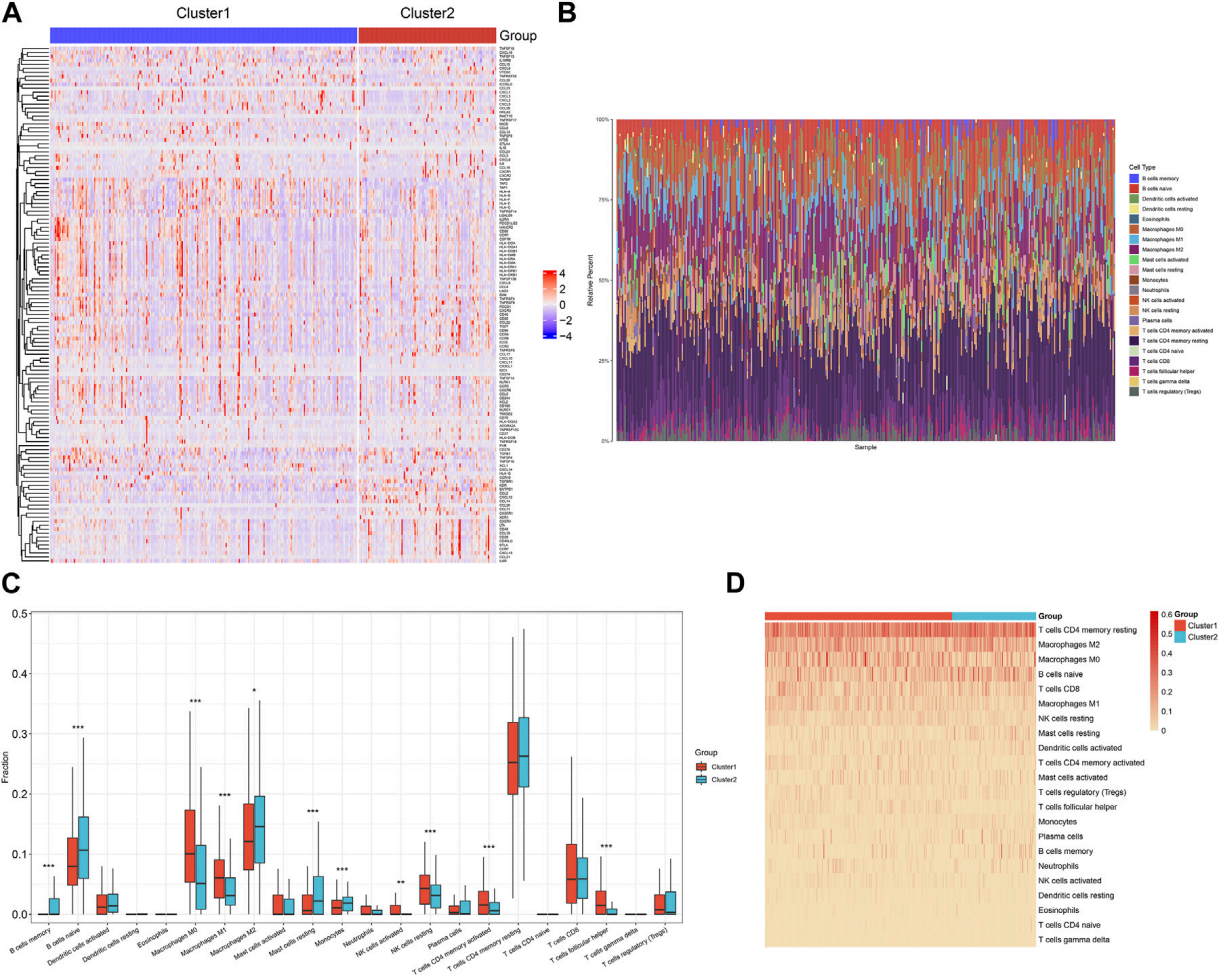
Tumor microenvironment heterogeneity across the two DNA damage repair-based subtypes in the TCGA-STAD cohort. **(A)** The transcriptional levels of immunomodulators across the two subtypes. Colors from blue to red denote low to high expression values of immunomodulators. **(B)** Landscape of the relative fractions of 22 immune cell types across TCGA-STAD. Each cell type is marked by unique color. **(C)** Comparison of the relative fractions of 22 immune cell types between subtypes. **(D)** The distribution of the relative fractions of 22 immune cell types across the two subtypes. The darker the color, the larger the relative fractions of immune cell types.

Then, we measured the transcriptional levels of immunomodulators (chemokines, receptors, MHC I, MHC II, immunostimulators, and immunoinhibitors) across TCGA-STAD. Notably, chemokines (CCL14, CCL2, CCL11, *etc.*), and immunostimulators (CD48, CD28, LTA, *etc.*) had higher levels in cluster2, while most MHC molecules displayed higher activity in cluster1 (Figure 3A). Through adopting CIBERSORT computational method, we computed the relative fractions of 22 immune cell types across TCGA-STAD (Figure 3B). In addition, the heterogeneity in immune cells between subtypes was assessed. In contrast to cluster1, cluster2 exhibited higher fractions of memory and naïve B cells, M2 macrophages, resting mast cells, and monocytes as well as lower fractions of M0 and M1 macrophages, activated and resting NK cells, activated memory CD4^+^ T cells, and follicular helper T cells (Figures 3C, D).

### Generation of a DNA damage repair-relevant signature for predicting clinical outcomes of gastric cancer

**FIGURE 4:**
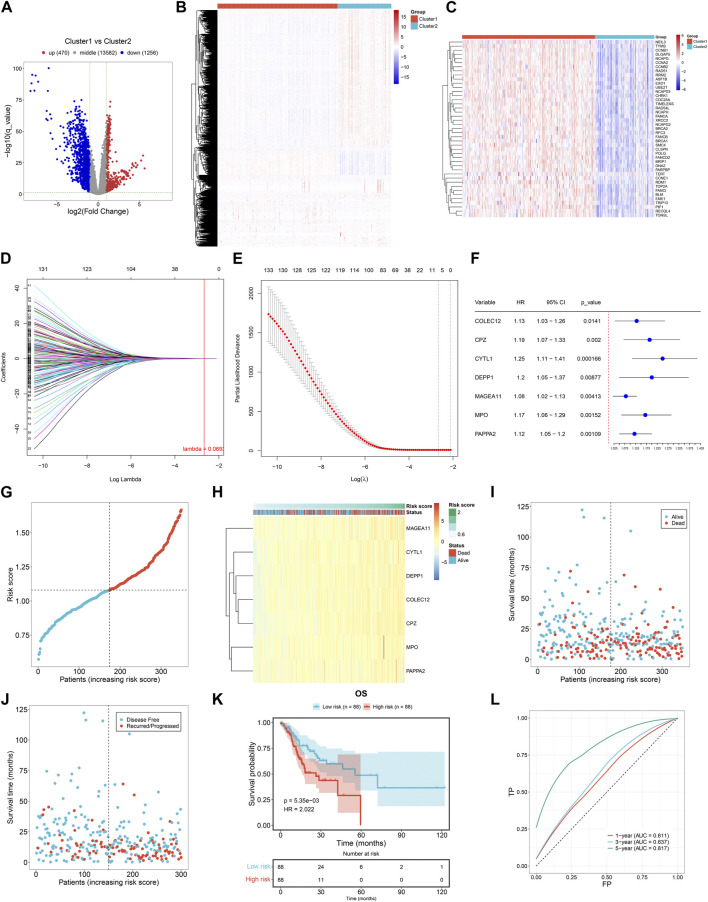
Generation of a DNA damage repair-relevant signature for predicting clinical outcomes of gastric cancer in the TCGA-STAD cohort. **(A)** Volcano diagram of the genes with differential expression in cluster1 *versus* cluster2 based upon the criteria of |FC|>2 and adjusted *p* < 0.05. Blue, downregulation; red, upregulation; grey, no significance. **(B)** Distribution of the transcription levels of above genes across the two DNA damage repair-based subtypes. Colors from blue to red denote low to high transcriptional levels. **(C)** Distribution of the transcription levels of DNA damage repair genes among them across the two subtypes. **(D)** LASSO coefficient profiling of prognostic DNA damage repair-relevant genes. The vertical dotted line shows the optimal lambda value. **(E)** Partial likelihood deviance in the LASSO gene signature through ten-fold cross-validation. The vertical dotted lines denote the optimal values based upon the minimum and 1-SE criteria. **(F)** Forest plot of the relationships of transcriptional levels of prognostic DNA damage repair-relevant genes with gastric cancer OS through univariate cox regression method. **(G)** Distribution of RiskScore across TCGA-STAD. Dotted lines show the median RiskScore as the grouping criteria. **(H)** Transcription levels of prognostic DNA damage repair-relevant genes across TCGA-STAD. Colors from blue to red denote low to high transcriptional levels. **(I)** Alive and dead status across patients with increasing RiskScore. **(J)** Disease free and recurred/progressed status across patients with increasing RiskScore. **(K)** K-M curves of OS between low- and high-risk cases in the training dataset. **(L)** ROC of survival status for the DNA damage repair-relevant RiskScore.

Totally, we determined 470 up- and 1256 downregulated genes in cluster1 *versus* cluster2 based upon the criteria of |FC|≥2 and adjusted *p* < 0.05 (Figures 4A, B). Among them, 43 were DNA damage repair genes, with notable upregulation in cluster1 (Figure 4C). Thereafter, the current study evaluated their prognostic implication in gastric cancer. With *p* ≤ 0.01, 138 DNA damage repair-relevant genes were significantly linked to OS of gastric cancer (Table 2), which were input into LASSO analysis for variable selection. TCGA-STAD samples were randomly and equally separated into training and test datasets. When the lambda value was 0.0693, and the regression coefficient was not equal to 0, seven DNA damage repair-relevant genes COLEC12, CPZ, CYTL1, DEPP1, MAGEA11, MPO, and PAPPA2 were finally selected (Figures 4D, E). Figure 4F depicts the univariate cox regression results of above genes. All of them acted as risky factors of gastric cancer OS. Based upon them, a DNA damage repair-relevant signature was generated, following the formula: RiskScore = 0.0103488452696626 * COLEC12 expression +0.0682893864499146 * CPZ expression + 0.0361252660190149 * CYTL1 expression + 0.04553168636357 * DEPP1 expression +0.0457508199288027 * MAGEA11 expression +0.00340309980118412 * MPO expression + 0.00350839302933862 * PAPPA2 expression. Figure 4G shows the distribution of RiskScore across TCGA-STAD. With the increase of RiskScore, the transcriptional level of COLEC12, CPZ, CYTL1, DEPP1, MAGEA11, MPO, and PAPPA2 gradually increased (Figure 4H). Based upon the median RiskScore, TCGA-STAD samples were classified as low- and high-risk groups. We observed less dead and recurred/progressed cases in low-than high-risk group (Figures 4I, J). In the training dataset, low-risk cases exhibited better OS outcomes (Figure 4K). ROC curves proved the significant superiority of this DNA damage repair-relevant RiskScore in predicting long-term OS outcomes with AUC at 5-year survival >0.8 (Figure 4L).

**TABLE 2:** Prognostic DNA damage repair-relevant genes with *p* ≤ 0.01 across TCGA-STAD.

Gene	P	HR	Gene	P	HR	Gene	P	HR
CYTL1	1E-04	1.261	LUM	0.003	1.210	COLEC11	0.006	1.164
GPX3	2E-04	1.241	PRSS23	0.003	1.253	DOK6	0.006	1.216
SLC7A2	3E-04	1.150	CHRDL1	0.003	1.086	IGFN1	0.006	1.100
PDE1B	5E-04	1.248	PLPPR4	0.004	1.178	CNRIP1	0.007	1.245
PLCL1	8E-04	1.29	ABCA6	0.004	1.172	BICC1	0.007	1.183
APOD	8E-04	1.120	PCDHB4	0.004	1.197	PCDHGA12	0.007	1.191
MPO	1E-03	1.172	MCC	0.004	1.230	NPR1	0.007	1.203
STK32A	0.001	1.151	MICU3	0.004	1.193	NRK	0.007	1.094
GLT8D2	0.001	1.264	SERPINF1	0.004	1.191	ELANE	0.007	1.129
SNCG	0.001	1.177	ADGRD1	0.004	1.133	DCLK1	0.007	1.126
GDF6	0.001	1.148	CRTAC1	0.004	1.109	FGF7	0.007	1.131
PAPPA2	0.001	1.117	DYNC1I1	0.004	1.151	EFS	0.007	1.185
GALNT15	0.001	1.176	PRKD1	0.004	1.201	RNF217	0.007	1.201
BCHE	0.001	1.111	FREM1	0.004	1.108	PDE2A	0.007	1.180
ARMCX1	0.001	1.242	NALCN	0.005	1.142	CNTN4	0.007	1.182
TCEAL7	0.001	1.208	PDE1A	0.005	1.197	LAMA2	0.007	1.163
EBF2	0.001	1.173	FRMD6	0.005	1.216	AKAP12	0.007	1.142
PAMR1	0.001	1.267	RBMS3	0.005	1.193	SLIT2	0.007	1.119
CPZ	0.001	1.197	FIBIN	0.005	1.185	CLIP4	0.008	1.196
EFEMP1	0.001	1.173	JAM3	0.005	1.211	DEPP1	0.008	1.199
PRICKLE1	0.001	1.214	MMRN1	0.005	1.132	AKT3	0.008	1.200
GNG11	0.002	1.310	MEOX2	0.005	1.111	RERG	0.008	1.131
KCNJ8	0.002	1.263	PLXDC2	0.005	1.198	DCN	0.008	1.172
GHR	0.002	1.171	CDO1	0.005	1.129	NAV3	0.008	1.148
RECK	0.002	1.262	C1QTNF2	0.005	1.188	PTGFR	0.008	1.142
CNTN1	0.002	1.110	HEYL	0.005	1.205	PLCXD3	0.008	1.094
ARMCX2	0.002	1.224	LRCH2	0.005	1.197	NCAM2	0.008	1.115
SVEP1	0.002	1.168	MAGEA11	0.005	1.075	DOK5	0.008	1.181
NUDT11	0.002	1.175	KLHL4	0.005	1.142	C11orf96	0.008	1.191
PIP4P2	0.002	1.268	FERMT2	0.005	1.178	FAM110B	0.008	1.156
CD36	0.002	1.183	LHFPL6	0.005	1.217	ASPA	0.009	1.141
EDNRA	0.002	1.244	BEX4	0.006	1.187	GLP2R	0.009	1.115
FBXL7	0.002	1.244	STEAP4	0.006	1.143	GEM	0.009	1.197
RTL8B	0.002	1.256	TF	0.006	1.078	ABCA8	0.009	1.101
RGS4	0.002	1.165	SLC22A17	0.006	1.170	ITGBL1	0.009	1.103
PHLDB2	0.002	1.197	FSTL1	0.006	1.240	GUCY1B1	0.009	1.202
KCNT2	0.002	1.185	RASSF8	0.006	1.173	FAM229B	0.009	1.202
ZNF521	0.003	1.243	CAV1	0.006	1.179	COPZ2	0.009	1.190
THSD7A	0.003	1.236	ABCA9	0.006	1.137	COLEC12	0.009	1.143
ERG	0.003	1.283	NAP1L2	0.006	1.119	ABCC9	0.010	1.141
FLRT2	0.003	1.159	MYB	0.006	0.864	GGT5	0.010	1.203
BRME1	0.003	0.817	MAGI2	0.006	1.200	ZFPM2	0.010	1.154
SORCS2	0.003	1.168	ROR2	0.006	1.146	SCUBE2	0.010	1.141
ITIH3	0.003	1.163	THPO	0.006	1.110	MID2	0.010	1.187
ADAMTS1	0.003	1.209	OMD	0.006	1.101	FABP4	0.010	1.093
IGFBP7	0.003	1.273	ASPN	0.006	1.142	HGF	0.010	1.168

Abbreviations: HR, hazard ratio.

### External verification of the DNA damage repair-relevant signature

**FIGURE 5:**
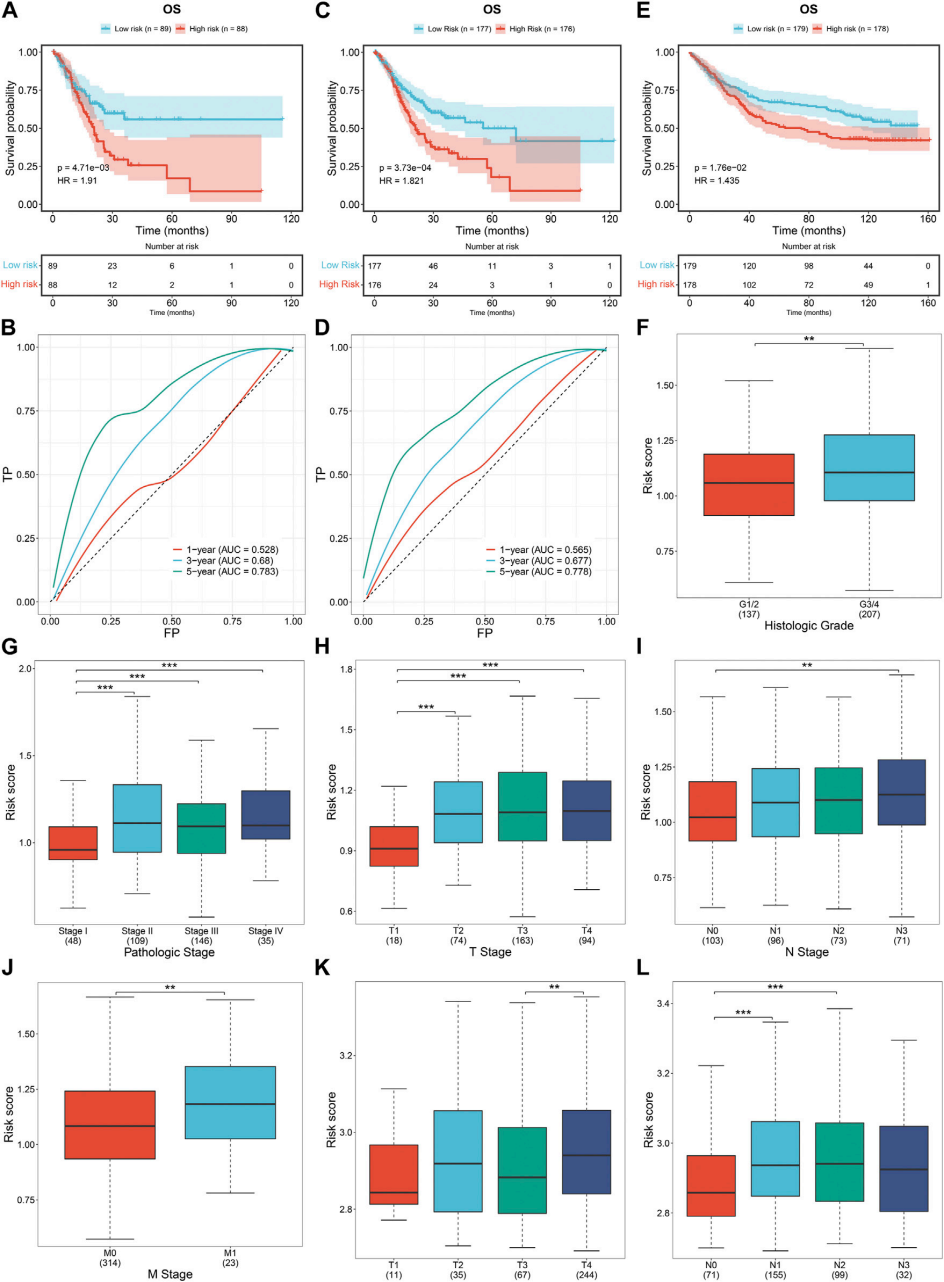
External verification of the DNA damage repair-relevant signature. **(A,B)** K-M curves of OS between low- and high-risk cases and ROC of survival status for this RiskScore in the test cohort. **(C,D)** K-M curves of OS between low- and high-risk cases and ROC of survival status for this RiskScore in the entire cohort. **(E)** K-M curves of OS between low- and high-risk cases in the GSE84433 cohort. **(F–J)** Distribution of RiskScore across distinct histological grade, pathological stage, and T, N, M stage across TCGA-STAD. **(K,L)** Distribution of RiskScore across distinct T, N stage in the GSE84433 cohort.

The predictive performance of the DNA damage repair-relevant signature was further verified. Both in the test and entire datasets, low-risk cases displayed more favorable OS outcomes in contrast to high-risk cases, with excellent performance in prediction of long-term survival (Figures 5A–D). The GSE84433 cohort was adopted for external verification, and the prognostic significance of this RiskScore were proven (Figure 5E). Higher RiskScore was observed in more advanced histological grade (Figure 5F), pathological stage (Figure 5G), and T, N, M stage (Figures 5H–J) across TCGA-STAD. The similar findings were found in the GSE84433 cohort (Figures 5K, L).

### Subgroup analysis of the DNA damage repair-relevant signature in prognosis prediction

**FIGURE 6:**
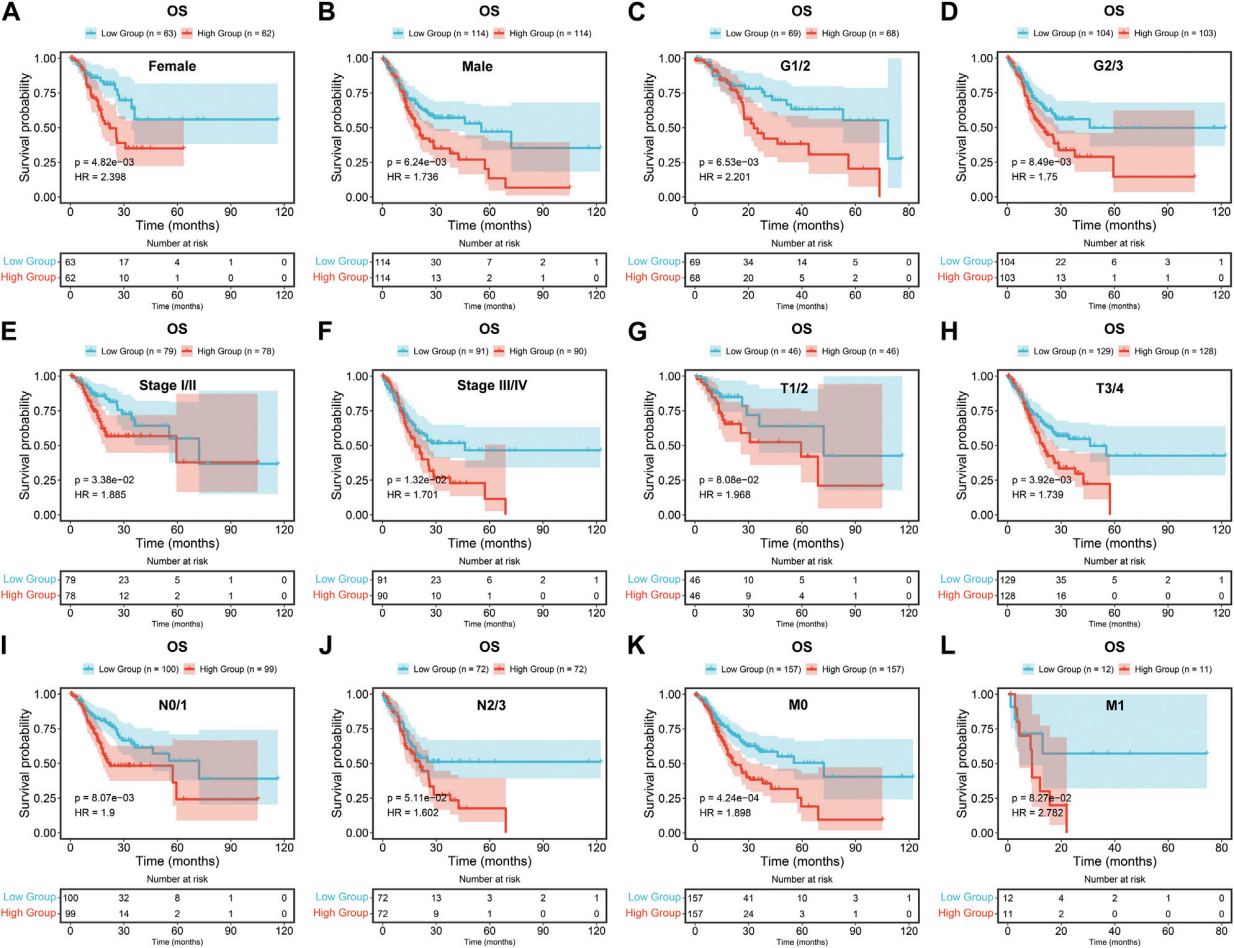
Subgroup analysis of the DNA damage repair-relevant signature in survival prediction in the TCGA-STAD dataset. **(A–L)** Prognostic differences of low- and high-risk patients in each subgroup stratified by conventional clinical parameters: sex, grade, stage, T, N, and M.

To assess the sensitivity of the DNA damage repair-relevant signature in predicting patient survival, we conducted subgroup survival analysis. Gastric cancer patients were stratified into distinct subgroups according to conventional clinical parameters: sex, grade, stage, T, N, and M (Figures 6A–L). As a result, in each subgroup, high-risk patients presented poorer survival outcomes *versus* low-risk patients, demonstrating the excellent sensitivity of the signature in survival prediction.

### Independency of the DNA damage repair-relevant signature in prognosis prediction and underlying molecular mechanisms

**FIGURE 7:**
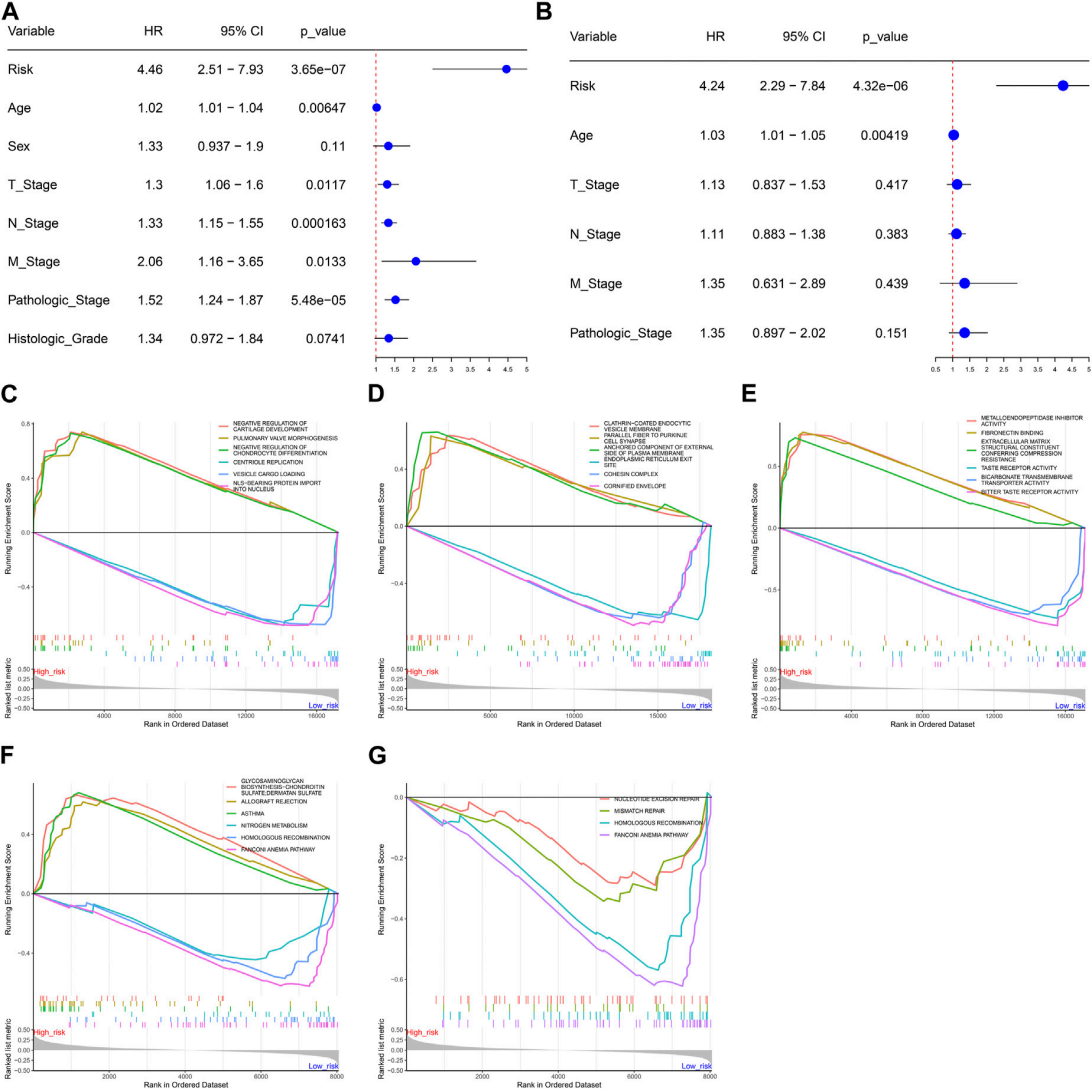
Independency of the DNA damage repair-relevant signature in prognosis prediction and underlying molecular mechanisms in the TCGA-STAD dataset. **(A,B)** Forest plots of the relationships of the DNA damage repair-relevant signature with OS *via* adopting uni- and multivariate cox regression methods. **(C–G)** GSEA of the enrichment levels of biological processes, cellular components, molecular functions, KEGG pathways, and DNA damage repair pathways in the low- and high-risk samples.

From uni- and multivariate cox regression results, the DNA damage repair-relevant signature together with age were independent risky factors of OS outcomes (Figures 7A, B). Molecular mechanisms underlying the signature were assessed through GSEA. For biological process, high RiskScore was positively linked to negative regulation of cartilage development and chondrocyte differentiation, and pulmonary valve morphogenesis, with negative relationships to centriole replication, vesicle cargo loading and NLS-bearing protein import into nucleus (Figure 7C). For cellular component, higher activity of clathrin-coated endocytic vesicle membrane, parallel fiber to purkinje cell synapse, anchored component of external side of plasma membrane was observed in high-risk cases, with lower activity of endoplasmic reticulum exit site, cohesion complex and cornified envelope (Figure 7D). For molecular function, high RiskScore exhibited positive relationships with metalloendopeptidase inhibitor activity, fibronectin binding, extracellular matrix structural constituent conferring compression resistance, with negative correlations to taste receptor activity, bicarbonate transmembrane transporter activity and bitter taste receptor activity (Figure 7E). In addition, we observed higher enrichment levels of immune-related pathways (glycosaminoglycan biosynthesis-chondroitin sulfate dermatan sulfate, allograft rejection and asthma) in high-risk cases, with lower enrichment levels of nitrogen metabolism, homologous recombination and Fanconi anemia pathway (Figure 7F). Also, the RiskScore was negatively correlated to DNA damage repair pathways (nucleotide excision repair, mismatch repair, homologous recombination and Fanconi anemia pathway) (Figure 7G).

### Relationships of the DNA damage repair-relevant signature with tumor microenvironment

**FIGURE 8:**
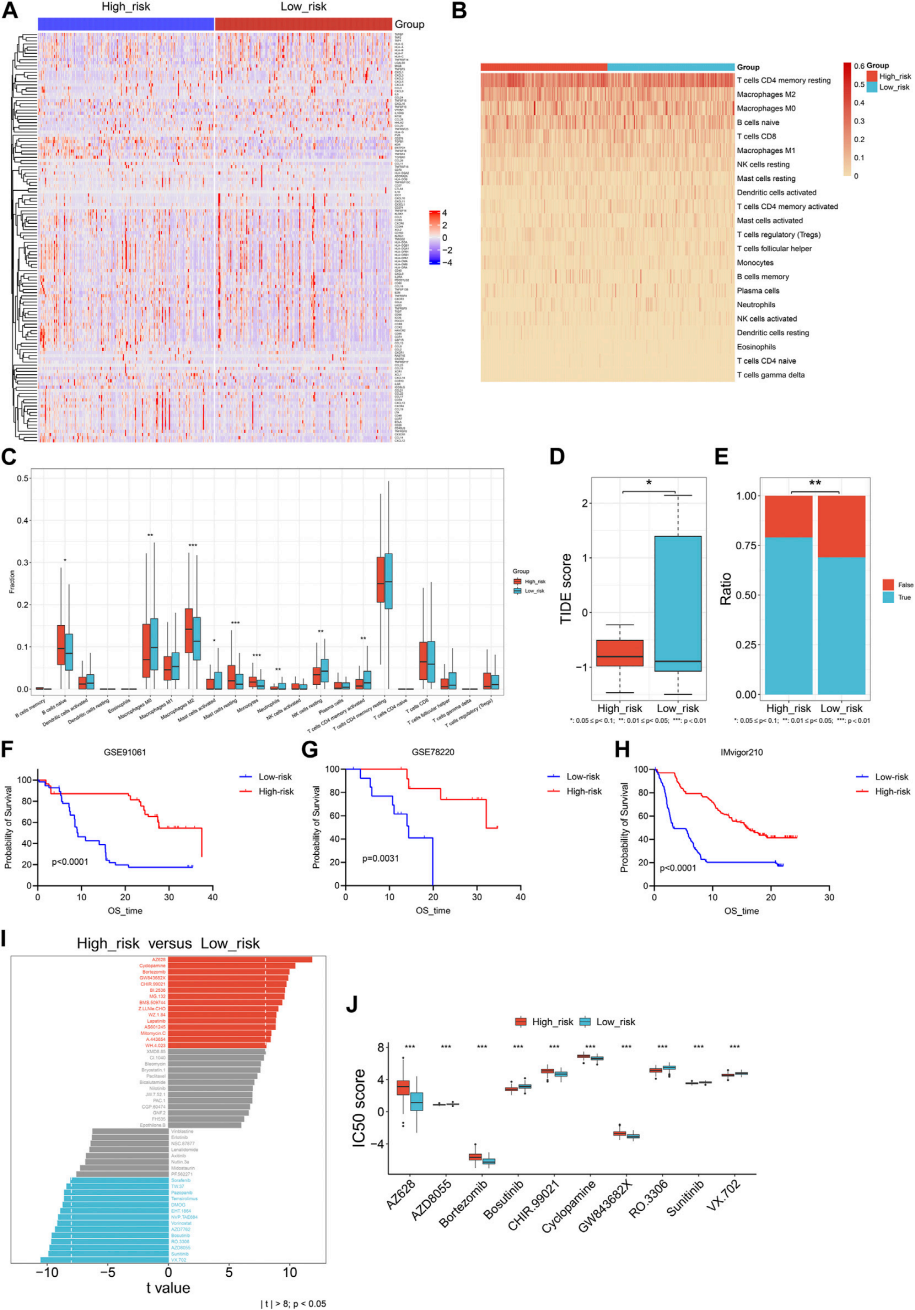
Relationships of the DNA damage repair-relevant signature with tumor microenvironment, immunotherapeutic response together with drug sensitivity across TCGA-STAD. **(A)** The transcriptional levels of immunomodulators in the low- and high-risk samples. Colors from blue to red represent low to high expression values of immunomodulators. **(B)** The distribution of the relative fractions of 22 immune cell types across the two groups. The darker the color, the larger the relative fractions of immune cell types. **(C)** Comparison of the relative fractions of 22 immune cell types between groups. **(D,E)** Comparisons of TIDE score and proportions of responders to immunotherapy between groups. **(F–H)** Prognostic differences of low- and high-risk patients in three immunotherapy cohorts: GSE91061, GSE78220 and IMvigor210. **(I)** Comparison of drug sensitivity in high-risk group *versus* low-risk group. **(J)** Box plot of the IC50 values of drugs between groups.

Next, relationships of the DNA damage repair-relevant signature with tumor microenvironment were assessed across TCGA-STAD. As illustrated in Figure 8A, High-risk cases exhibited higher transcriptional levels of immunostimulators (CD276, ENTPD1, TNFSF18, TNFSF4, LTA, *etc.*), and chemokines (CCL14, CCL17, CCL22, *etc.*) in contrast to low-risk cases. Higher infiltration of naïve B cells, M2 macrophages, resting mast cells, monocytes, as well as lower infiltration of M0 macrophages, resting NK cells, activated memory CD4 T cells were observed in the high-risk samples (Figures 8B, C). Above findings unveiled the heterogeneity in tumor microenvironment between low- and high-risk groups.

### Relationships of the DNA damage repair-relevant signature with immunotherapeutic response and drug sensitivity

TIDE computational approach was adopted for inferring immunotherapeutic response. As a result, lower TIDE score was investigated in high-risk patients (Figure 8D), and this subpopulation had higher proportions of responders to immunotherapy (Figure 8E). Three independent immunotherapy cohorts: GSE91061, GSE78220, and IMvigor210 were collected for further evaluating the efficacy of the DNA damage repair-relevant signature in predicting immunotherapeutic response. It was shown that high-risk patients had better survival outcomes in comparison to low-risk patients after immunotherapy (Figures 8F–H), demonstrating that high-risk patients had the higher possibility to benefit from immunotherapy. In addition, low-risk group displayed lower IC50 scores of AZ628, Bortezomib, CHIR.99021, Cyclopamine, and GW843682X, as well as higher IC50 scores of AZD8055, Bosutinib, RO.3306, Sunitinib, and VX.702 compared with high-risk group (Figures 8I, J).

### Experimental verification of the DNA damage repair-relevant genes

**FIGURE 9:**
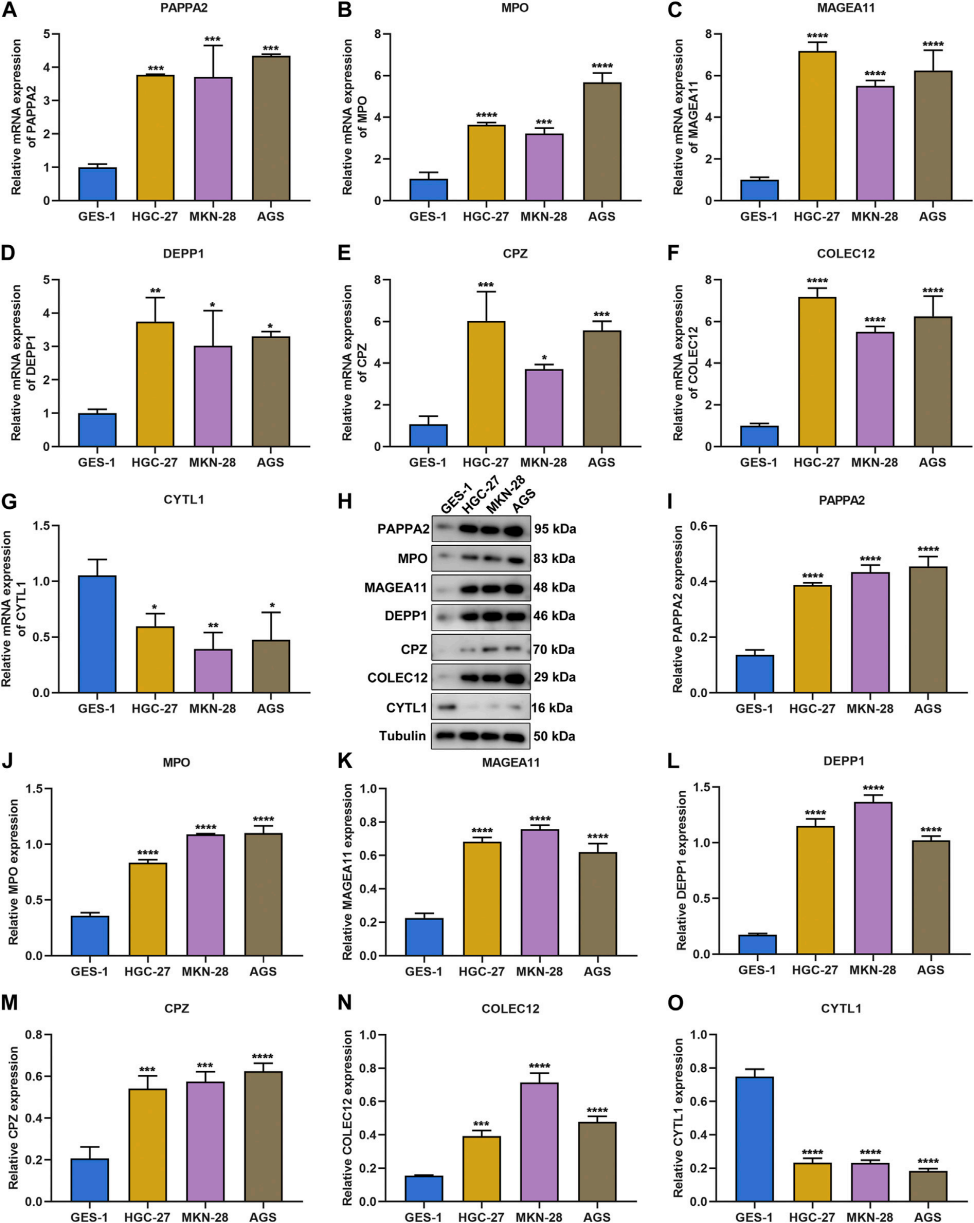
Experimental verification of the DNA damage repair-relevant genes. **(A–G)** RT-qPCR of the transcriptional levels of PAPPA2, MPO, MAGEA11, DEPP1, CPZ, COLEC12, and CYTL1 in GES-1, HGC-27, MKN-28 and AGS cell lines. **(H–O)** Immunoblotting of the protein levels of PAPPA2, MPO, MAGEA11, DEPP1, CPZ, COLEC12, and CYTL1 in GES-1, HGC-27, MKN-28, and AGS cell lines.

The DNA damage repair-relevant genes were further experimentally verified. In contrast to gastric mucosa epithelial cell line GES-1, transcriptional levels of PAPPA2, MPO, MAGEA11, DEPP1, CPZ, and COLEC12 were higher in gastric cancer cell lines HGC-27, MKN-28 and AGS, with lower transcriptional level of CYTL1 (Figures 9A–G). The consistent results were observed at the protein level (Figures 9H–O).

### Silencing CYTL1 facilitates intracellular ROS accumulation and impairs migrative capacity of gastric cancer cells

**FIGURE 10:**
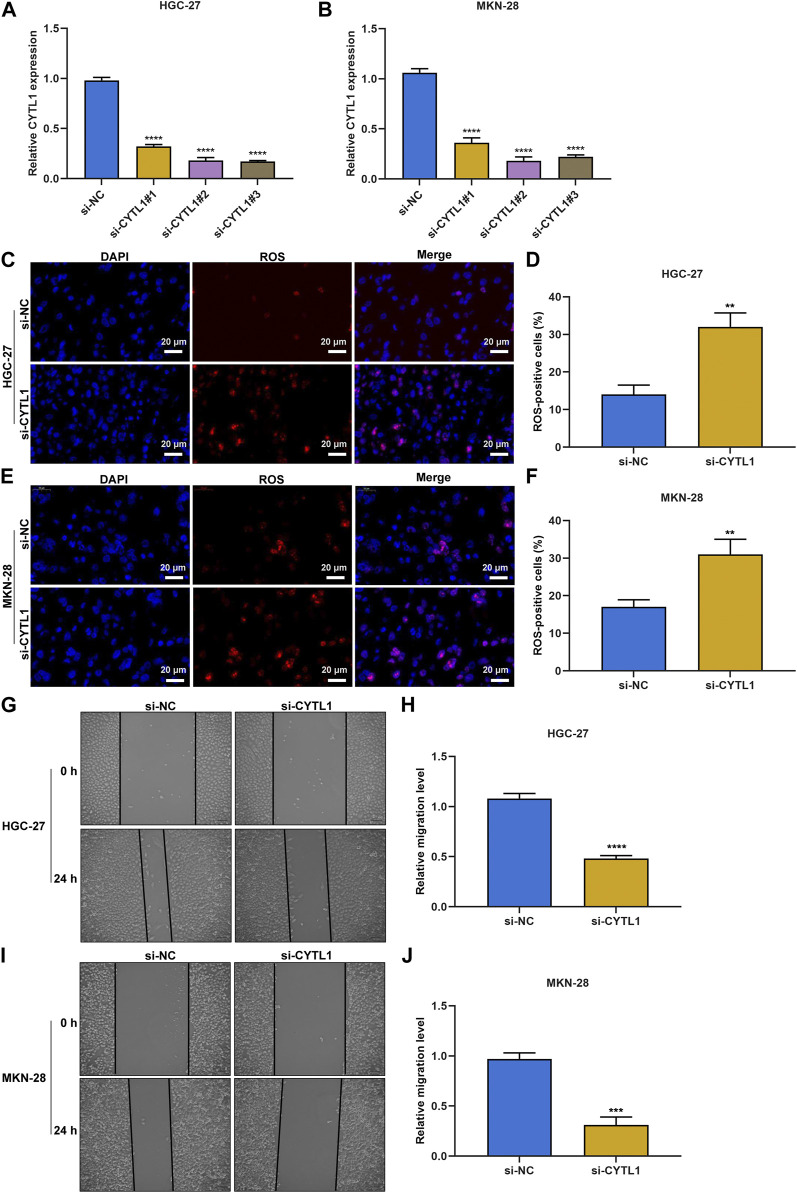
Silencing CYTL1 facilitates intracellular ROS accumulation and impairs migrative capacity of gastric cancer cells. **(A,B)** RT-qPCR of the expression of CYTL1 in HGC-27 and MKN-28 cells transfected with siRNAs targeting CYTL1. **(C–F)** ROS fluorescence probe detecting intracellular ROS in transfected HGC-27 and MKN-28 cells. Bar, 20 μm. **(G–J)** 0-h and 24-h photographs of wound healing experiment for transfected HGC-27 and MKN-28 cells. Bar, 50 μm.

Among the DNA damage repair-relevant genes, the biological significance of CYTL1 in gastric cancer remains indistinct. CYTL1 expression was notably silenced by its specific siRNAs both in HGC-27, and MKN-28 gastric cancer cells (Figures 10A, B). According to ROS fluorescence probe detection, we found that CYTL1-silenced HGC-27, and MKN-28 cells presented the higher accumulation of ROS (Figures 10C–F). In addition, migrative capacity of HGC-27, and MKN-28 cells was remarkably impaired by CYTL1 knockdown (Figures 10G–J).

## Discussion

Gastric cancer remains a primary reason of global cancer mortality, with limited therapeutic regimens as well as undesirable survival (Wang et al., 2021). The heterogeneity remains a challenge to clinical management, and the current molecular classification system for gastric cancer primarily covers genomic, molecular together with morphological characteristics (Kumar et al., 2022). The current research proposed a novel DNA damage repair-based subtyping, which might be a suitable complement to existing molecular classification system. Notably, there was a notable heterogeneity in prognostic stratification, tumor microenvironment and somatic mutations between DNA damage repair-based subtypes.

Most DNA damage repair genes exhibited upregulation and MSI subtype had higher ratios in cluster1 *versus* cluster2, indicating the DNA damage repair activating and inhibitory status of cluster1 and cluster2, respectively. In contrast to cluster1, better OS outcomes were found in cluster2. In addition, there was extensive heterogeneity in clinicopathological traits between subtypes. Somatic mutation is required for the development and growth of gastric cancer (Sethi et al., 2020). More frequent somatic mutation occurred in cluster1, especially SYNE2, RYR1, TTN, NEB, DOCK3, AHNAK2, EPHA5, DNAH8, FAT3, and SSPO. The heterogeneity in immune cells within the tumor microenvironment was investigated between subtypes, with cluster2 exhibiting higher fractions of memory and naïve B cells, M2 macrophages, resting mast cells, and monocytes as well as lower fractions of M0 and M1 macrophages, activated and resting NK cells, activated memory CD4^+^ T cells, and follicular helper T cells. Previous research has demonstrated that DNA damage repair alterations influence M2 polarization of macrophages to remodel the tumor microenvironment (Meng et al., 2019). NK cell exhaustion owing to sustained proliferation leads to impaired NK cell functions with loss of cytokine generation and lytic activity. Activation of DNA damage repair can ameliorate NK cell exhaustion (Alvarez et al., 2019).

Advanced patients display undesirable prognostic outcomes, with the median survival < 1 year (Li et al., 2022). Hence, developing novel agents is urgently required for improving the overall survival rate. Despite immunotherapy as a therapeutic regimen for specific subtypes of gastric cancer, the heterogeneity is still a key barrier to the development of potent agents (Wang et al., 2021). Herein, the DNA repair-relevant signature enabled to infer pharmacogenomic landscape across gastric cancer. High-risk cases were more likely to respond to immunotherapy based upon higher TMB score and lower TIDE score. Consistently, in three independent immunotherapy cohorts, high-risk cases presented more favorable survival outcomes, and had the higher possibility to benefit from immunotherapy. Moreover, low-risk cases were more sensitive to AZ628, Bortezomib, CHIR.99021, Cyclopamine, and GW843682X, with higher sensitivity to AZD8055, Bosutinib, RO.3306, Sunitinib, and VX.702. Among these therapeutic compounds, Bortezomib in synergy with other chemotherapeutic agents can improve the therapeutic effects against gastric cancer (Bae et al., 2008; Zhang et al., 2020). Cyclopamine sensitizes TRAIL-resistant gastric cancer cells to TRAIL-induced apoptosis through endoplasmic reticulum stress-induced upregulation of death receptor 5 and survivin degradation (Na et al., 2017). A combined treatment of docetaxel and sunitinib has a synergistic antitumor effect in a preclinical model, but the combined regimen cannot significantly prolong survival of patients with metastatic gastric cancer in a phase II clinical trial (Yi et al., 2012).

This study experimentally verified the levels of DNA repair-relevant genes, with higher levels of PAPPA2, MPO, MAGEA11, DEPP1, CPZ, and COLEC12 and lower level of CYTL1 in gastric cancer cells *versus* controls. Limited evidence has proven the functions of above DNA repair-relevant genes in gastric cancer. PAPPA2 is in relation to prognostic outcomes of gastric cancer together with immunes cells in the tumor microenvironment (Qin et al., 2021). Epidemiological studies demonstrate that MPO polymorphism influences the risk of gastric cancer (Zhu et al., 2006; Steenport et al., 2007). MPO, a DNA repair-relevant biomarker, is induced by alcohol with prognostic implication in gastric cancer (Zhang et al., 2018). COLEC12 can integrate *H. pylori* infection, PGE2-EP2/4 pathway as well as innate immunity in gastric diseases (Chang et al., 2018). CYTL1 is a hub gene linked to the pathogenesis and prognostic outcomes of gastric cancer (Nie et al., 2020). Our further experiments demonstrated that silencing CYTL1 enabled to facilitate intracellular ROS accumulation and impair migrative capacity of gastric cancer cells, indicative of CYTL1 as a possible therapeutic target of gastric cancer.

Non-etheless, a few limitations of this study needed to be acknowledged. Since the datasets we used were from distinct high-throughput sequencing platforms, intratumoral or intra-patient heterogeneity may be inevitable. Moreover, despite the discovery of the roles of DNA damage repair in prognosis and tumor microenvironment in gastric cancer, biological mechanisms underlying these phenomena remain indistinct. Therefore, large-scale prospective cohorts as well as function and mechanism experiments are required for validating and explaining the roles of DNA damage repair in gastric cancer. In addition, the median RiskScore was utilized for classifying gastric cancer cases into low- and high-risk groups. Non-etheless, the optimal cutoff of RiskScore will be a preferable strategy for stratifying patients.

## Conclusion

In summary, our research proposed two DNA damage repair subtypes with distinct clinical outcomes, somatic mutation together with tumor microenvironment traits, and found that the DNA damage repair-relevant signature could robustly predict clinical outcomes of gastric cancer and was correlated to tumor microenvironment, immunotherapeutic response, and sensitivity to small molecular compounds, which might become a useful tool for survival prediction and therapeutic guidance for gastric cancer. Thus, this comprehensive research of DNA damage repair genes will assist to comprehend their roles and significance in gastric cancer.

## Data Availability

The datasets presented in this study can be found in online repositories. The names of the repository/repositories and accession number(s) can be found in the article/Supplementary Material.

## Abbreviations

CDF, cumulative distribution function; CTLs, cytotoxic T lymphocytes; FC, fold change; GO, Gene Ontology; HR, hazard ratio; K-M, Kaplan–Meier; KEGG, Kyoto Encyclopedia of Genes and Genomes; LASSO, least absolute shrinkage and selection operator; OS, overall survival; PCA, principal component analysis; ROC, receiver operating characteristic; TIDE, Tumor Immune Dysfunction and Exclusion; TMB, tumor mutation burden.
